# Sitting for Too Long, Moving Too Little: Regular Muscle Contractions Can Reduce Muscle Stiffness During Prolonged Periods of Chair-Sitting

**DOI:** 10.3389/fspor.2021.760533

**Published:** 2021-11-03

**Authors:** Alexander R. Kett, Thomas L. Milani, Freddy Sichting

**Affiliations:** ^1^Department of Human Locomotion, Institute of Human Movement Science and Health, Chemnitz University of Technology, Chemnitz, Germany; ^2^Research & Development, Mercedes-Benz AG, Böblingen, Germany

**Keywords:** prolonged sitting, muscle stiffness, muscle contraction, sedentary behavior, back muscles, electrical stimulation, biomechanics

## Abstract

In modern Western societies, sedentary behavior has become a growing health concern. There is increasing evidence that prolonged sitting periods can be associated with musculoskeletal disorders. While it is generally recognized that back muscle activity is low during chair-sitting, little is known about the consequences of minor to no muscle activity on muscle stiffness. Muscle stiffness may play an important role in musculoskeletal health. This study investigated the effects of regular muscle contractions on muscle stiffness in a controlled experiment in which participants sat for 4.5 h. Neuromuscular electrical stimulation in the lumbar region of the back was applied to trigger regular muscle contractions. Using stiffness measurements and continuous motion capturing, we found that prolonged sitting periods without regular muscle contractions significantly increased back muscle stiffness. Moreover, we were able to show that regular muscle contractions can prevent those effects. Our results highlight the importance of consistent muscle activity throughout the day and may help explain why prolonged periods of chair-sitting increase the susceptibility to common pathological conditions such as low back pain.

## Introduction

Common sedentary behaviors include office work, driving automobiles, using public transportation, and screen time. In modern Western societies, those behaviors can sum up to a sitting time of 8.4–9.3 h per day (Healy et al., 2011; Clemes et al., 2014; van der Velde et al., 2017). Recent research related to the coronavirus disease 2019 (COVID-19) indicate that sedentary behavior time has further increased since the beginning of the pandemic, particularly in people who are now working from home (Fukushima et al., 2021; Wilke et al., 2021). Those prolonged periods of sitting are considered as an independent risk factor for health, including an increased risk of developing metabolic and chronic cardiovascular diseases (Hamilton et al., 2007; Healy et al., 2011) and increased mortality (Chau et al., 2013; Stamatakis et al., 2019). Further research associates long sitting periods with musculoskeletal disorders, such as increased muscle stiffness (Kett and Sichting, 2020), fatigue (Callaghan and McGill, 2001; van Dieën et al., 2001), discomfort (Sammonds et al., 2017; Cardoso et al., 2018; Waongenngarm et al., 2020), and, at worst, low back pain (Porter and Gyi, 2002; Gupta et al., 2015; Lunde et al., 2017). Notably, low back pain is a growing public health concern in modern Western societies and a tremendous socioeconomic burden (Lis et al., 2007; Manchikanti et al., 2009; Hartvigsen et al., 2018).

A recent study by Raichlen et al. (2020) on sitting behavior among the Hadza, a hunter-gatherer population, sheds new light on the association between sedentary behavior and musculoskeletal disorders (Raichlen et al., 2020). Interestingly, the hunter-gatherers show similar periods of inactivity (9.9 h per day) compared to industrialized populations (Raichlen et al., 2020). However, associated musculoskeletal disorders are scarce among non-industrialized populations (Volinn, 1997; Lopez et al., 2006). The lower level of musculoskeletal disorders among non-industrialized populations might be related to a greater level of physical activity. Another possible explanation for the discrepancy in musculoskeletal disorders might be the style of rest during periods of inactivity. While industrialized populations often sit on chairs, sedentary postures among hunter-gatherers include kneeling, squatting, and ground-sitting (Pontzer et al., 2010; Raichlen et al., 2020). Raichlen et al. (2020) showed that those postures, particularly squatting, require higher muscle activity levels than chair-sitting (Raichlen et al., 2020). Based on these findings, it seems reasonable to question the general association between sedentary behavior and musculoskeletal disorders. One can hypothesize that our bodies are not well-built for spending much of our day sitting in chairs with minor to no muscle activity (O'Keefe et al., 2010; Hamilton, 2018; Raichlen et al., 2020).

Previous research has shown that prolonged periods of chair-sitting result in increased passive back muscle stiffness (Kett and Sichting, 2020). It has been suggested that the low level of muscle activity during chair-sitting, and the static nature of the sitting postures causes a restriction of the muscle metabolism, with adverse effects on blood flow, muscle tissue oxygenation, and regulation of inflammation (McGill et al., 2000; Valachi and Valachi, 2003; Visser and van Dieën, 2006; Kell and Bhambhani, 2008). Further, the reduced muscle metabolism appears to trigger a reactive imbalance in the muscle cell (McGill et al., 2000; Kell and Bhambhani, 2008), promoting spontaneous formations of weak but long-lasting cross-bridges between myosin heads and actin filaments (Hill, 1968; Campbell and Lakie, 1998). Subsequently, passive muscle stiffness increases (Simons and Mense, 1998; Proske and Morgan, 1999). If this theoretical framework proves to be true, intervention strategies that elicit dynamic muscle contractions during chair-sitting should counter an increase in passive muscle stiffness (Hsueh et al., 1997; Campbell and Lakie, 1998) by improving muscle metabolism (Saltin et al., 1998; Crenshaw et al., 2006).

This study aims to provide experimental evidence for the above-mentioned theoretical framework. Using surface electrical stimulation of lower back muscles during prolonged periods of chair-sitting allows us to stimulate back muscles at a sensory and motor threshold level (Hultman et al., 1983; Maffiuletti et al., 2011). When using low-amplitude currents, electrical stimulation is perceived through somatic sensory receptors mainly located in cutaneous and subcutaneous tissues (termed sensory threshold). Thus, electrical stimulation at the sensory threshold does not trigger muscle contractions directly (Purves et al., 2004; Maffiuletti et al., 2008). In contrast, when applying current amplitudes above the sensory threshold (termed motor threshold), an increasing number of efferent terminal axon branches are excited and result in contractile protein interaction (Hultman et al., 1983; Maffiuletti et al., 2008). Previous studies have shown that surface electrical stimulation above the sensory threshold is an effective tool for stimulating lumbar muscles (Kim et al., 2016; Sions et al., 2019). Comparing the effects of surface electrical stimulation at the sensory and motor threshold on the lower back's passive muscle stiffness during a 4.5-h sitting period will help us to test the general hypothesis that intervention strategies that elicit muscle activity during chair-sitting counter an increase in passive muscle stiffness. We predict that stimulation at the motor threshold will diminish increases in passive muscle stiffness. In contrast, we predict that electrical stimulation of the back muscles at the sensory threshold will not affect passive muscle stiffness. Spinal kinematics will be recorded during all measurements to monitor the possible effects of sitting posture and postural variation on passive muscle stiffness during the multiple sitting periods.

## Materials and Methods

### Participants

Fifteen volunteers (seven women and eight men) participated in this study. The volunteers were employees or students at the university. All participants (age: 28.9 ± 5.0 years, weight: 74.5 ± 10.3 kg, height: 176.9 ± 10.0 cm) were required to be healthy, with no current injuries or conditions that would cause sitting abnormalities or prohibit the application of surface electrical stimulation. Further, all participants had to pause moderate and high physical activities 24 h before the experiment to avoid possible muscle fatigue and altered muscle stiffness. Each participant gave written informed consent to participate in the study. The study was approved by the institutional ethics committee of the Faculty of Behavioral and Social Sciences at Chemnitz University of Technology (approval number: V-370-17-FS-E.-Stimulation-07022020) and conducted in accordance with the Declaration of Helsinki.

### Intervention Strategies and Settings

**Figure 1:**
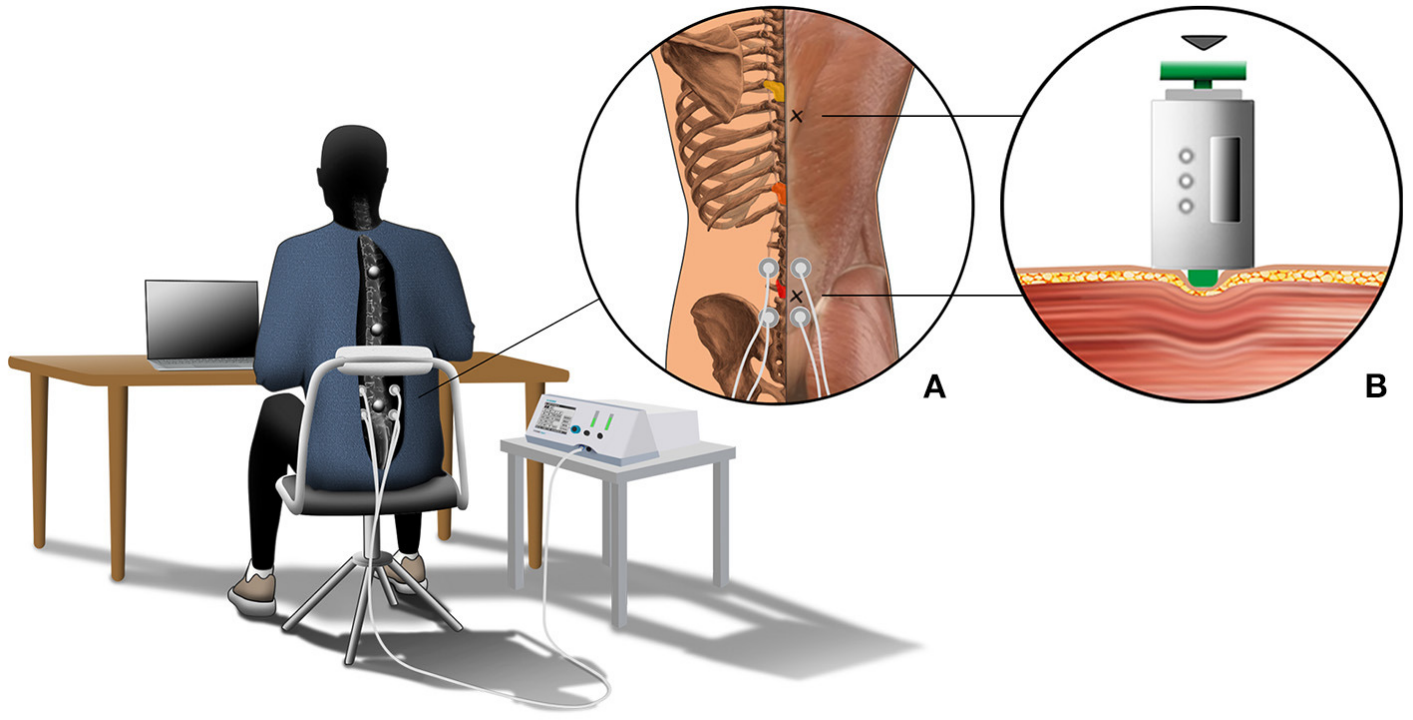
The graphical depiction illustrates the experimental setup. All participants sat for 4.5 h at a desk on a height-adjustable chair to complete their regular office activities. The chair's back cushion was removed, leaving only the metal frame cushioned with foam to guarantee marker visibility. Further, all participants had to wear a long-sleeve T-shirt with a cut-out at the back exposing the spinal area. Motion data of the back were captured using three retro-reflective skin markers, which were placed on the spinous processes of vertebrae T5, T10, and L2. The three vertebrae are colored in yellow (T5), orange (T10), and red (L2) in close-up **(A)**. Further, close-up **(A)** shows the locations of the stiffness measurement (marked with an x) and details about the NMES condition. Here, two electrodes were placed on the left and right sides of the lumbar spine. Close-up **(B)** shows details about the passive muscle stiffness measurement. Muscle stiffness was defined by the slope of the relationship between indentation depth and resistance force.

We used neuromuscular electrical stimulation (NMES) applied by a portable stimulator (PHYSIOMED-Expert; PHYSIOMED Elektromedizin AG, Schnaittach, Germany) at the lumbar region of the back to test the effect of regularly induced muscle contractions on passive muscle stiffness. In total, we tested three conditions for each participant: CONTROL (sitting without NMES), NMES_SENSOR_ (stimulation with low-amplitude currents, where electrical stimulation is perceived through somatic sensory receptors mainly located in cutaneous and subcutaneous tissues), and NMES_MOTOR_ (electrical stimulation with greater current amplitudes, where an increasing number of efferent terminal axon branches are excited). We used NMES_MOTOR_ to test the effect of regular muscle contractions on muscle stiffness, and NMES_SENSOR_ to test for placebo effects of the electrode application. The skin was disinfected before electrode placement. Following the motor point map by Behringer et al. (2014), we placed two electrodes (electrode diameter: 3.2 cm, inter electrode distance: 5 cm) on the left and right side of the lumbar spine (Figure 1) (Behringer et al., 2014). We chose a frequency-modulated current for the NMES_SENSOR_ and NMES_MOTOR_ conditions (pulse shape: triangular biphase, stimulation frequency: 7–14 Hz, contraction time = 1 ms, rest time: 70–142 ms, duration: 5 min) (Tucker et al., 2010). To determine the individual current amplitude for the NMES_SENSOR_ condition, we followed the protocol proposed by Maffiuletti et al. (2011). The participants had to lay relaxed in a prone position. Following electrode positioning and instructions, current amplitude was progressively increased by the investigator from zero to the point of current perception, when the participant indicated initial (lowest) perception of stimulus sensation (tingling, itching, heat). The respective current amplitude was defined as the sensory threshold. After reaching the sensory threshold, the current was reduced to zero again. Threshold determination was repeated twice at each side of the lumbar spine, and the average current was used as the individual sensory threshold. Among all participants, the average sensory threshold current was 3.6 ± 1.3 mA. For the NMES_MOTOR_ condition, the motor threshold was defined as three times the individual sensory threshold, following Kantor et al. (1994). When the calculated current amplitude exceeded 16.1 mA (current density, which the manufacturer declares not to exceed), we used 16.1 mA as the motor threshold. Among all participants, the average motor threshold current was 9.7 ± 2.5 mA.

### Experimental Protocol

Each participant completed three sitting periods of 4.5 h each within 10 days to test the three conditions (CONTROL, NMES_SENSOR_ and NMES_MOTOR_). The order of the conditions was randomized for each subject. Each sitting period started between 7:00 and 8:00 a.m. Participants sat at a desk on a height-adjustable chair to conduct their regular office activities (e.g., reading and writing documents, laptop computer work) (Figure 1). Kinematic data were collected for periods of 15 min throughout the 4.5 h sitting period. Between the intervals, short breaks (<5 min) were allowed, e.g., to use the restroom. Stiffness data of the back muscles were collected before and after the sitting period. One examiner collected all kinematic and stiffness data and supervised the NMES. For the NMES_SENSOR_ and NMES_MOTOR_ conditions, NMES was applied at the sensory or motor threshold for 5 min, followed by a 10-min recovery phase. The first electrical stimulation started after 15 min and was applied 17 times during the 4.5-h sitting period.

### Stiffness Measurement

We measured the muscle's resistance against deformation as a surrogate measure for muscle stiffness (Simons and Mense, 1998; Wilke et al., 2018) using a custom-built indentometer device. The handheld device was used in a previous study to non-invasively investigate back muscle stiffness (Kett and Sichting, 2020). A prior study by Wilke et al. (2018) on the gastrocnemius muscle indicates an excellent test-retest reliability (intraclass correlation coefficient: 0.84) for the indentometer device (Wilke et al., 2018). As described by Kett and Sichting (2020), the device contains a load cell (Compression Load Cell FX1901, TE Connectivity, Schaffhausen, Switzerland) and a membrane potentiometer (ThinPot 10 kOhm, Spectra Symbol, Salt Lake City, USA) to measure the resistance force and displacement of a circular indentation probe (Ø 11.3 mm) (Kett and Sichting, 2020). As depicted in Figure 1B, the probe was placed two centimeters to the right lateral side of the lumbar and thoracic spine to measure the muscles alongside the spine. For the stiffness measurements, the participants lay down in a relaxed prone position. Each measurement consisted of three consecutive indentations, where the investigator compressed the tissue up to a defined indentation depth. The indentation depth was 12 mm for the muscles alongside the lumbar spine. The indentation depth at the muscles alongside the thoracic spine was 8 mm. The corresponding force of resistance was recorded to calculate the passive muscle stiffness.

### Acquisition and Analysis of Kinematic Data

Motion data were captured at 30 Hz using an eight-camera motion analysis system (Vicon Motion Systems Ltd., Oxford, United Kingdom). Three retro-reflective skin markers (diameter: 16.0 mm) were placed on the spinous processes of the vertebrae T5 (thoracic spine), T10 (thoracic spine), and L3 (lumbar spine) (Figure 1) (Claus et al., 2009; Korakakis et al., 2014) to quantify three-dimensional motions of the back. We modified the chair's backrest and participant's garment to guarantee the markers' continuous visibility (Figure 1). Data processing was performed using Vicon Nexus 2.8.1 (Vicon Motion Systems Ltd, UK) and R Studio (R Foundation for Statistical Computing, Vienna, Austria). Motion capture data were downsampled to 1 Hz, and a recursive fourth-order Butterworth low-pass filter (5 Hz cutoff frequency) was used to process the kinematic data.

The thoracolumbar angle (θ_TH_), calculated as the angle between T5, T10, and L3, was used to evaluate sitting posture. Further, sample entropy (SampEn), a time series regularity measure, was used to evaluate postural variation. According to (Delgado-Bonal and Marshak, 2019), SampEn measures with a tolerance r the regularity of patterns similar to a given template of a given length (further defined as m) (Delgado-Bonal and Marshak, 2019). The continuously recorded θ_TH_ was used for the time series analysis. A lower value of SampEn during a given sitting period indicates more self-similarity in the time series and, thereby, a lower postural variation. Based on protocols from previous postural control studies, *m* = 2 was utilized and a tolerance of *r* = 0.1^*^SD was chosen (Søndergaard et al., 2010; Lubetzky et al., 2018). The Package “TSEntropies” in R Studio was used to compute SampEn.

### Data Analysis and Statistics

All statistical analyses were carried out using IBM SPSS Statistics, version 25 (IBM, Armonk, New York, USA). Means and standard deviations (mean ± SDs) were calculated for the stiffness/kinematic data, and a Shapiro–Wilk test of normality was performed. Day-to-day variability (interday coefficient of variation, CV%) has been analyzed for the stiffness measurements before the sitting period on the three days of data recording. A two-way repeated ANOVA was used for normally distributed data to analyze the impact of sitting time and conditions on back muscle stiffness of the lumbar and thoracic spine. When a significant main effect between conditions (CONTROL, NMES_SENSOR_ and NMES_MOTOR_) and/or time (measurement before and after the sitting period) was observed, a Bonferroni-adjusted *post-hoc* analysis was performed.

Further, we performed one-way repeated ANOVAs for normally distributed data to test whether sitting posture (mean spinal curvature) and postural variation (SampEn) were different between the three conditions (CONTROL, NMES_SENSOR_ and NMES_MOTOR_). If a significant main effect was observed between conditions, a Bonferroni-adjusted *post-hoc* analysis was performed. The level of significance was set at α = 0.05 for all statistical tests.

## Results

**Figure 2:**
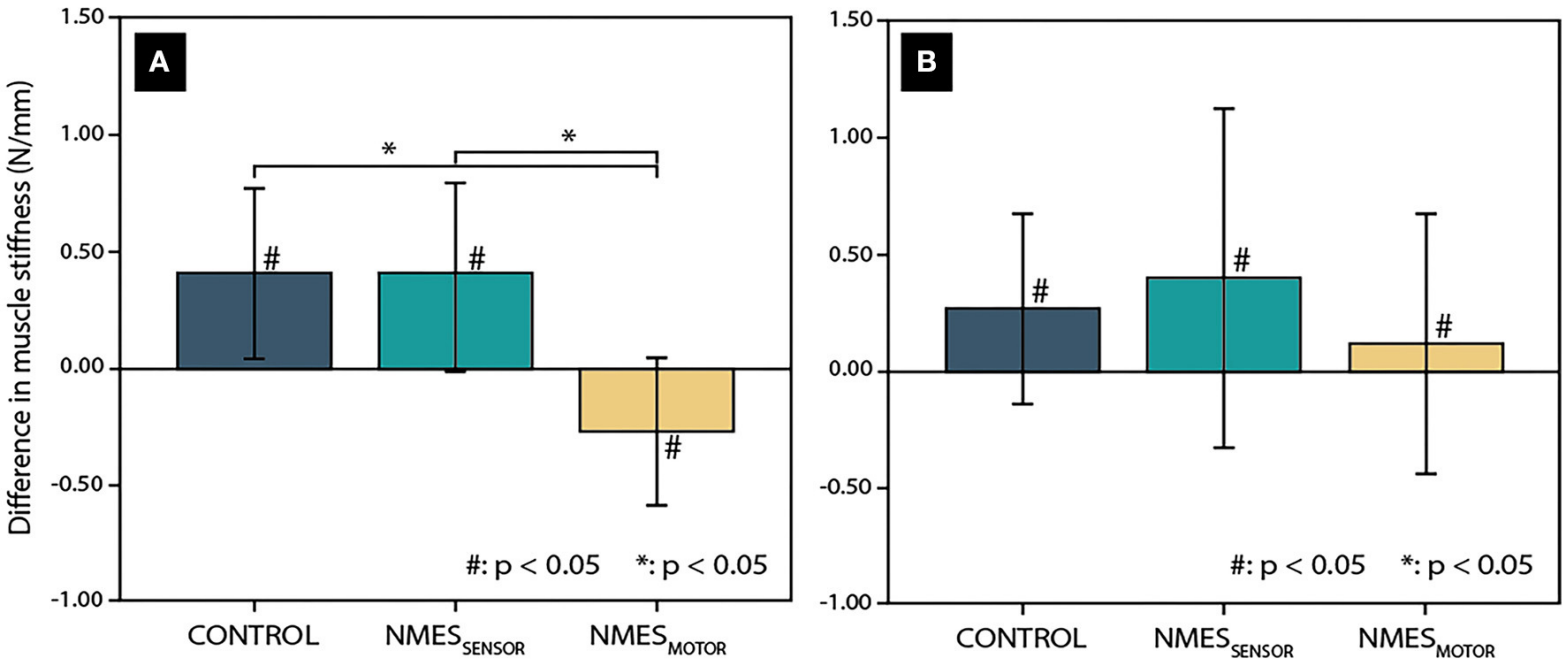
Change in muscle tissue stiffness after a 4.5-h sitting period in the lumbar spine **(A)** and thoracic spine **(B)** for the three conditions (CONTROL: without neuromuscular electrical stimulation, NMES_SENSOR_: neuromuscular electrical stimulation at the sensory threshold, and NMES_MOTOR_: neuromuscular electrical stimulation at the motor threshold.) Significant differences related to the sitting period are indicated by a hash sign (#), while an asterisk (*) indicates significant differences between the tested conditions.

The day-to-day variability was 9.8 and 16.6% for the lumbar and thoracic muscle stiffness measurements. Further, the two-way repeated ANOVA indicated no significant differences between the initial stiffness measurements (before sitting) on the 3 days of data recording. Changes in lumbar and thoracic muscle stiffness after the 4.5-h sitting period are presented in Figure 2 for each condition (CONTROL, NMES_SENSOR_, and NMES_MOTOR_). Lumbar and thoracic muscle stiffness increased significantly for CONTROL (lumbar: +16.5%, pre: 2.5 ± 0 .5 vs. post: 2.9 ± 0.5 N/mm, *p* < 0.01; thoracic: +9.4%, pre: 2.9 ± 0.6 vs. post: 3.2 ± 0.6 N/mm, *p* = 0.02), and NMES_SENSOR_ (lumbar: +17.6%, pre: 2.3 ± 0.5 vs. post: 2.7 ± 0.5 N/mm, *p* = 0.02; thoracic: +12.8%, pre: 3.2 ± 0.8 vs. post: 3.6 ± 0.9 N/mm *p* = 0.045). For NMES_MOTOR_, lumbar muscle stiffness decreased significantly by −10.8% (pre: 2.5 ± 0.6 vs. post: 2.2 ± 0.5 N/mm, *p* = 0.06), but thoracic muscle stiffness did not change significantly (+4.1%, pre: 3.1 ± 0.6 vs. post: 3.2 ± 0.8 N/mm, *p* = 0.36).

Among the conditions, changes in lumbar muscle stiffness differed significantly (Figure 2). NMES_MOTOR_ was significantly different from CONTROL and NMES_SENSOR_ (*p* < 0.01, respectively). In contrast, no significant difference was found between CONTROL and NMES_SENSOR_ (*p* = 0.73). No significant differences were found between the NMES conditions for the changes in thoracic muscle stiffness (Figure 2).

Besides muscle stiffness, we analyzed sitting posture and postural variation to test for differences between the conditions. During the 4.5-h sitting period, the average θ_TH_ was 164.1 ± 3.7° for CONTROL, 165.0 ± 4.6° for NMES_SENSOR_, and 165.6 ± 4.2° for NMES_MOTOR_. A one-way repeated ANOVA revealed no significant differences between the conditions. Similarly, no statistically significant differences were found for postural variation. On average, SampEn was 0.3 ± 0.1 for CONTROL, 0.3 ± 0.1 for NMES_SENSOR_, and 0.3 ± 0.1 for NMES_MOTOR_.

## Discussion

This study aimed to assess the importance of regular muscle activity during prolonged chair-sitting. We hypothesized that regular muscle contractions could counter an increase in passive back muscle stiffness. To test the hypothesis, we applied bouts of electrical stimulation to the lumbar back area—both at a sensory and a motor threshold level. The most important finding was that the stimulation at the motor threshold level during a 4.5-h sitting period led to a significant decrease in back muscle stiffness. Another important result was that the stimulation at the sensory threshold level showed no effect on back muscle stiffness. Similar to the control condition (no electrical stimulation involved), we found a significant increase in back muscle stiffness. These results add to the growing body of literature recognizing the importance of regular muscle activity during daily sedentary behaviors (Hamilton, 2018; Kuster et al., 2020; Raichlen et al., 2020).

Consistent with previous findings (Kett and Sichting, 2020), the control condition showed a significant increase in back muscle stiffness of +16.5% in the lumbar spine and of +9.4% in the thoracic spine after a prolonged sitting period of 4.5 h. While the mechanisms for this sitting-related effect remain unclear, most hypotheses revolve around reduced metabolism in muscle tissue due to the low activity of postural muscles during predominantly static chair-sitting postures (Valachi and Valachi, 2003; Visser and van Dieën, 2006; Akkarakittichoke and Janwantanakul, 2017; Raichlen et al., 2020). However, this study did not measure any indicator of muscle tissue metabolisms, such as blood flow or inflammation markers directly, several lines of evidence indicate that the vicious circle of restricted microcirculation and increased muscle stiffness is most pronounced in the often-preferred slump sitting posture. Slumped sitting is characterized by an excessive posterior tilt of the pelvis and decreased lumbar spine lordosis (Claus et al., 2009; Nairn et al., 2013). It is proposed that this posture relies mainly on the passive lumbopelvic structures (e.g., spinal ligaments) to maintain a resting sitting position. Following this argument, previous research has shown that the activity level seems to be lowest during slump sitting (Claus et al., 2009; Mörl and Bradl, 2013; Nairn et al., 2013). Our motion analysis revealed that the average thoracolumbar angle (θ_TH_) was about 165°. Under the assumption that a θ_TH_ of 180° represents a flat sitting posture, the participants in our study likely spent most of their sitting time in a slump sitting posture (θ_TH_ < 180°). However, caution is required here, since a detailed analysis of sitting postures requires the calculation of angles at the thoracic, thoracolumbar, and lumbar regions. Such an approach was used, for example, by Claus et al. (2009). Despite the uncertainty about the degree of slump sitting, our motion analysis showed that the sitting postures and postural variabilities were generally similar between the three tested conditions. These results strengthen confidence in our findings on the effects of electrical stimulation on back muscle stiffness.

The most prominent finding to emerge from the electrical stimulation interventions is that stimulation at the motor threshold level led to a significant decrease in stiffness of the lumbar back muscles of −10.8% after the 4.5-h sitting period. This finding is likely related to regular muscle contractions. When applying current amplitudes at a motor threshold level, an increasing number of efferent terminal axon branches are excited and result in contractile protein interaction (Hultman et al., 1983; Maffiuletti et al., 2008; Sions et al., 2019). It may be that the rhythmic muscle contractions evoked by the electrical stimulation mimicked the naturally acting blood and lymph pump and thereby enhanced the microcirculation in the muscle tissue (Levine et al., 1990; Pittman, 2000; Tucker et al., 2010). Here we speculate that these processes led to maintenance or restoration of the physiological muscle tissue metabolism, preventing an imbalance in the muscle cell and consequent formations of long-lasting cross-bridges between myosin heads and actin filaments. A similar argument was provided by Hsueh et al. (1997), who showed that electrical stimulation at a motor threshold level reduced the muscle stiffness in muscles with myofascial trigger points (Hsueh et al., 1997). One somewhat unexpected finding of our study was that stiffness of the lumbar back muscles dropped below the baseline measurement after the prolonged sitting period of 4.5 h. The result suggests that the electrical stimulation at the motor threshold level not only compensates for increased muscle stiffness but further promotes muscle relaxation, similar to massage interventions (Kett and Sichting, 2020). Another interesting result was that we found significant effects of the electrical stimulation for the lumbar region but not for the thoracic region. This result may be explained by the fact that NMES recruits muscle zones close to the electrode. The recruitment diminishes proportionally with increasing distance from the electrode (Vanderthommen et al., 2000). Although the results of the thoracic measurements indicate a trend toward a reduced increase in muscle stiffness, considerably more research is required to develop a complete picture of muscle tissue response caudal and cranial to the stimulated area.

In contrast to stimulation at the motor threshold level, our stimulation at the sensory threshold level did not affect lumbar back muscle stiffness. Despite regular stimulation, muscle stiffness increased by 17.6% in the lumbar spine and 12.8% in the thoracic spine over the 4.5-h sitting period. This finding is consistent with a previous study by Hsueh et al. (1997), who also found no effect of electrical stimulation at the sensory level on muscle stiffness. A possible explanation for this result may be that electrical stimulation at the sensory threshold level is perceived through somatic sensory receptors mainly located in cutaneous and subcutaneous tissues (Purves et al., 2004). Thus, the low-amplitude currents likely do not trigger muscle contractions (Maffiuletti et al., 2008).

To gain more confidence in our findings, further research could investigate the effects of electrical stimulations in more detail and address some limitations. It would be of interest to identify the muscles of the lower back that were recruited by the electrical stimulation. Here, this study leaves some uncertainties. Further, the precise mechanism which explains the decrease in muscle stiffness in response to impulses at the motor threshold level remains to be analyzed. In this regard, accompanying blood flow measurements are strongly recommended. The effects of electrical stimulation on blood flow are currently limited to lower body muscles (Levine et al., 1990; McNeil et al., 2006; Tucker et al., 2010). Another question that remains to be answered is how to translate the muscle response elicited by the electrical stimulation into regular movements and voluntary contractions. For this study, we applied low-frequency electrical stimulation, which seems comparable to muscle activities during moderate aerobic exercises at 60–70% of the peak heart rate (Deley et al., 2005). However, considerably more work needs to be done to determine the contraction forces elicited by stimulation at the motor threshold level relative to maximum voluntary contractions. To quantify muscle contractions, a study similar to this one should be carried out using assessment techniques that are independent of electrical signals between the nerve and muscle, including laser doppler myography (Scalise et al., 2013; Casaccia et al., 2015), acoustic myography (Harrison et al., 2013; Harrison, 2018), or piezoresistive sensors (Esposito et al., 2018).

Notwithstanding these limitations, our study seems to support the Inactivity Missmatch Hypothesis proposed by Raichlen et al. (2020). They suggest that human physiology is adapted to more consistent muscle activity throughout the day associated with a combination of both moderate-to-vigorous physical activity and sedentary time spent in active rest postures. In this regard, Raichlen et al. (2020) showed that resting postures in hunter-gatherers involve increased muscle activity that is greater than chair-sitting sedentary postures used in industrialized populations (Raichlen et al., 2020). Although these findings are limited to electromyographic measurements of leg muscles, they align with a growing body of literature, which agrees on generally low muscular activity during chair-sitting (Claus et al., 2009; van Dieën et al., 2009). We add to these findings by providing the first experimental evidence that regular contractions of lumbar back muscles during prolonged chair-sitting can counter an increase in passive muscle stiffness.

Albeit this study used electrical stimulation to mimic regular bouts of increased muscular activity, the results support the evidence-based guidelines for frequent active breaks during prolonged periods of chair-sitting (Thorp et al., 2014; Waongenngarm et al., 2018). Another important practical implication of this study is that NMES revealed its potential as an intervention strategy for people forced to engage in prolonged periods of chair-sitting, such as professional drivers or people with disabilities. A future study could assess the long-term effects of electrical stimulation at the motor threshold level during prolonged sitting periods on low back pain development. Another question raised by this study is whether populations that spend most of their sedentary time in active rest postures, like the Hadza (Raichlen et al., 2020), would show a minor increase in back muscle stiffness. Following these avenues would be a fruitful area for further work. It might help gain a broader understanding of musculoskeletal disorders associated with chair-sitting sedentary postures used in industrialized populations.

## Data Availability Statement

The raw data supporting the conclusions of this article will be made available by the authors, without undue reservation.

## Ethics Statement

The studies involving human participants were reviewed and approved by Institutional Ethics Committee of the Faculty of Behavioral and Social Sciences at Chemnitz University of Technology. The patients/participants provided their written informed consent to participate in this study.

## Author Contributions

AK, FS, and TM designed the study, analyzed, interpreted the data, and wrote the manuscript. AK and FS collected and processed the data and prepared all figures. All authors contributed to the article and approved the submitted version.

## Funding

The publication of this article was funded by Chemnitz University of Technology.

## Conflict of Interest

AK received his salary from the Mercedes-Benz AG. However, Mercedes-Benz AG was not involved in the design and execution of the study, in the collection, analysis, and interpretation of the data, or in the preparation, review, and approval of the manuscript. The remaining authors declare that the research was conducted in the absence of any commercial or financial relationships that could be construed as a potential conflict of interest.

## Publisher's Note

All claims expressed in this article are solely those of the authors and do not necessarily represent those of their affiliated organizations, or those of the publisher, the editors and the reviewers. Any product that may be evaluated in this article, or claim that may be made by its manufacturer, is not guaranteed or endorsed by the publisher.
